# Right Ventricular Functional Improvement after Pulmonary Rehabilitation Program in Patients with COPD Determined by Speckle Tracking Echocardiography

**DOI:** 10.5935/abc.20180123

**Published:** 2018-09

**Authors:** Batur Gonenc Kanar, Ipek Ozmen, Elif Ozari Yildirim, Murat Ozturk, Murat Sunbul

**Affiliations:** 1 Marmara University, Istanbul - Turquia; 2 Sureyyapasa Chest Medicine Research and Training Hospital, Istanbul - Turquia

**Keywords:** Ventricular Dysfunction, Right / rehabilitation, Pulmonary Disease, Chronic Obstructive / rehabilitation, Echocardiography / methods, Strain, Speckle Tracking

## Abstract

**Background:**

Although right ventricular (RV) dysfunction in pulmonary diseases has been associated with increased morbidity, tools for RV dysfunction identification are not well defined.

**Objective:**

The aim of this study was to evaluate the magnitude of RV dysfunction by means of speckle tracking echocardiography (STE) in patients with chronic obstructive pulmonary disease (COPD) and to investigate whether STE could be used as an index of RV improvement after a pulmonary rehabilitation (PR) program.

**Methods:**

Forty-six patients with COPD undergoing PR program and 32 age-sex matched healthy subjects were enrolled. RV function was evaluated at admission and after PR program by conventional two-dimensional echocardiography (2DE) and STE. In addition, exercise tolerance of subjects was evaluated using the six-minute walk test (6MWT).

**Results:**

COPD patients had worse RV function according to STE and 2DE as well. STE was more sensitive than conventional 2DE in determining RV improvement after PR program - RV global longitudinal strain (LS): 20.4 ± 2.4% vs. 21.9 ± 2.9% p < 0.001 and RV free wall LS: 18.1 ± 3.4% vs. 22.9 ± 3.7%, p < 0.001). RV free wall LS was directly related to distance walked at baseline 6MWT (r = 0.58, p < 0.001) and to the change in the 6MWT distance (6MWTD Δ) (r = 0.41, p = 0.04).

**Conclusions:**

We conclude that STE might be as effective as 2DE for evaluation of global and regional RV functions. STE may become an important tool for assessment and follow-up of COPD patients undergoing PR program to determine the relationship between RV function and exercise tolerance.

## Introduction

The right ventricle plays an important role in the morbidity and mortality of patients with signs and symptoms of cardiopulmonary disease.^1^ Although transthoracic two-dimensional echocardiography (2DE) provides important information about the right ventricular (RV) anatomy and function, the RV complex geometry and crescent-shaped structure wrapped around the left ventricle (LV) make accurate assessment difficult.^2^ Moreover, conventional 2DE measures, including velocity and displacement-based analyses, can be affected by translational motion of the heart and respiratory variation. The new echocardiographic method of speckle tracking echocardiography (STE) assesses myocardial deformation on grayscale (B-mode) images and can be used to evaluate both global and regional myocardial strain without being limited by the Doppler beam angle.^3,4^

Patients with advanced chronic respiratory disease regularly experience distressing symptoms despite optimal pharmacological treatment. Pulmonary rehabilitation (PR) complements conventional medical therapy, and has been clearly demonstrated to reduce dyspnea, increase exercise performance, and improve RV functions.^5^ Today, is well known that chronic obstructive pulmonary disease (COPD) patients experience substantial mortality and morbidity from RV function impairment.^6,7^

A number of studies have used conventional 2DE to evaluate the RV in patients with cardiopulmonary diseases, but there is relatively limited information concerning the assessment of RV performance by means of speckle tracking-derived strain.^8,9^ Therefore, we sought to analyze the use of STE in the assessment of global and regional RV function and impact of PR program on it.

## Methods

### Study design and participants

Subjects were recruited from Sureyyapasa Chest Medicine and Thoracic Surgery Research and Training Hospital, Istanbul, Turkey. Fifty-seven patients with moderate-to-very severe COPD (Global Initiative for Chronic Obstructive Lung Disease, GOLD classes 2-4) were enrolled in the study. Six patients were excluded from the analyses due to the poor quality of their echocardiographic records. In the remaining patients, the apical segment of the RV free wall and the apical septum could not be analyzed in 3 and in 2 patients, respectively.

All patients had a previous diagnosis of symptomatic COPD. The control group included 32 healthy volunteers. Patients with impairment of LV systolic function (ejection fraction < 55%), significant valvular heart disease, cardiomyopathy, history of coronary artery disease, and malignancy were excluded. The investigation complies with the principles outlined in the Declaration of Helsinki. The study was approved by the local Ethics Committee and written informed consent was obtained from all participants.

Adult patients with COPD with medically optimized symptomatic lung disease, admitted to the outpatient PR program, were referred by respiratory physicians after an initial multidisciplinary assessment clinic with a respiratory or rehabilitation physician, cardiology physician, nurse, and physiotherapist. Before starting the PR program, we obtained medical histories and performed physical examination of all patients. Specific measurements recorded at the beginning and end of PR program included 6-minute walk test (6MWT), mMRC (modified Medical Research Council) dyspnea scale, the BODE index - body mass index (BMI), degree of obstruction (FEV1), dyspnea (mMRC scale), exercise capacity. The PR program consisted of 2 sessions each day for 6 days per week for a total of 4 weeks. Each session lasted 30 minutes and included symptom-limited exercise training (walking or cycling).

All 6MWTs were performed on a flat surface, enclosed, temperature-controlled corridor using standardized instructions.^10,11^ Two 6MWTs and echocardiographic examinations were performed at the beginning of the pre-rehabilitation and post-rehabilitation assessments at the end of PR program due to possible learning effect. The best 6MWT was recorded and used for analysis. The 6MWTDΔ (delta) was determined by the difference between pre- and post-rehabilitation of 6MWTs. The effect of the 6MWT after the PR program was evaluated by BODE index and the mMRC score.

### Conventional and speckle-tracking echocardiography

All echocardiographic examinations of patients and healthy controls were performed in accordance with the American Society of Echocardiography guidelines using an ultrasound system (IE33, Philips Medical Systems, Andover, MA, US).^12^ Estimation of systolic pulmonary artery pressure (sPAB) was based on tricuspid regurgitation peak velocity using the simplified Bernoulli equation: 4x(tricuspid regurgitation peak velocity)^2^+ right atrial pressure (RAP). Estimation of RAP was done on the basis of the inferior vena cava diameter and collapse index.^2^ Tricuspid annular plane systolic excursion (TAPSE) is defined as the total excursion of the tricuspid annulus from end-diastole to end-systole, and it is measured typically at the lateral annulus using M-mode Isovolumic relaxation time (IVRT), isovolumic contraction time (IVCT), myocardial performance index (MPI) (calculated as [IVRT + IVCT]/ejection time), and ejection time intervals were measured using either pulsed-wave Doppler (PWD) or Doppler tissue imaging (DTI) at the lateral tricuspid annulus. RV and LV ejection fractions from 2D methods were calculated as (end-diastolic volume - end-systolic volume)/end-diastolic volume.

**Figure 1:**
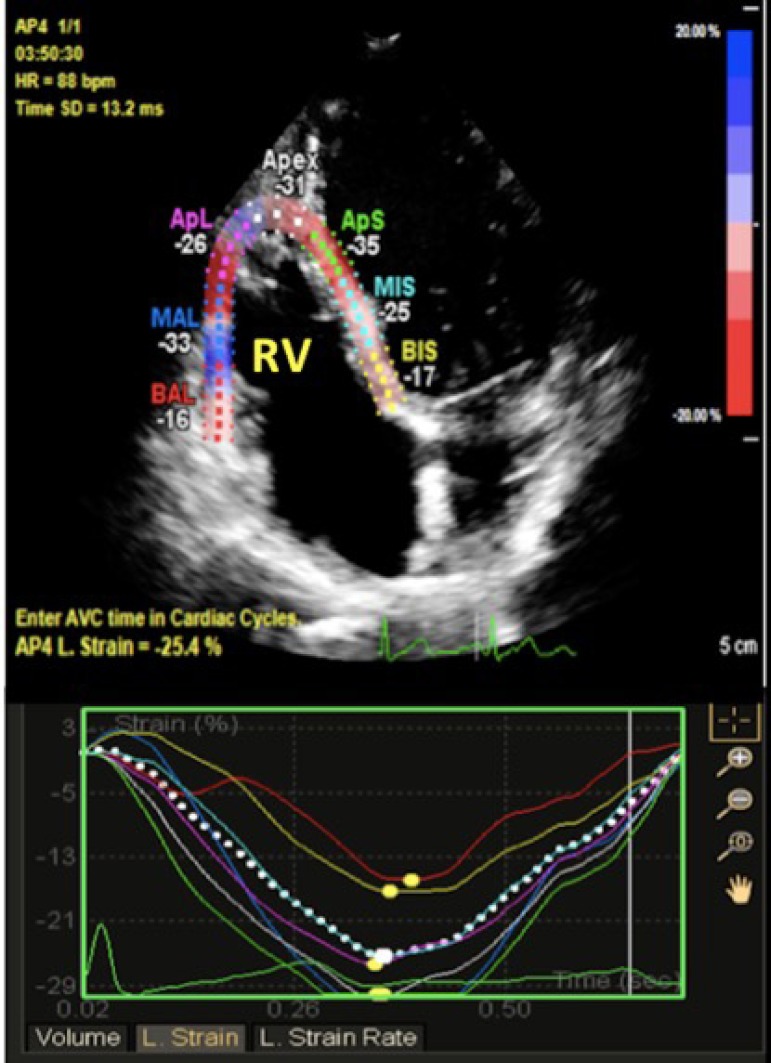
Representative two-dimensional right ventricular strain images. Speckle-tracking apical four chamber view showing global and regional right ventricular longitudinal strain. L. Strain: Longitudinal strain.

The general principles that underlie 2D speckle-tracking modalities have been previously described.^13,14^ 2D echocardiographic grayscale apical 4-chamber images and a frame rate of 70 to 80 frames/s were obtained, which seems to be the best compromise between appropriate temporal resolution and acceptable spatial definition of the LV lateral wall and RV free wall. In postprocessing analysis, the region of interest was obtained by tracing the RV endocardial borders at the level of the septum and the free wall in a still frame at end-systole. An automated software program calculated the frame-to-frame displacements of speckle pattern within the region of interest throughout the cardiac cycle. Longitudinal strain (LS) curves were obtained from six RV segments (basal, mid, and apical segments of the RV free wall and septum); the global RV strain curve was based on the average of the six regional strain curves, and longitudinal strain curves of the lateral LV wall were obtained by repeating the same analysis (Fig. 1). The extent of myocardial deformation (defined as global or regional longitudinal strain) was expressed as a percentage of the longitudinal systolic shortening compared with diastolic shortening for each segment of interest. All analyses were repeated twice one day later by the same observer in order to assess intraobserver variability, which was calculated as the average difference between the 10 measurements taken. A second independent observer repeated the analyses for the assessment of interobserver variability, which was calculated as the absolute difference divided by the average of the two observations of all parameters. The intraobserver and interobserver variability were 5% and 7 %, respectively.

### Statistical analysis

All statistical tests were performed with a commercially available software program (SPSS 16.0 for Windows; SPSS, Inc., Chicago, IL, USA). The variables were investigated using visual (histograms, probability plots) and analytical methods (Kolmogorov-Smirnov/Shapiro-Wilk test) to determine whether or not they are normally distributed. In sample size calculation, 46 COPD patients and 32 healthy subjects in each group would be needed to detect a 2-point difference in DAN scale, with a power of 80% and 1% of significance level. Categorical variables are presented as numbers and percentages and continuous data expressed as mean ± standard deviation. Since all variables were normally distributed, correlation coefficients and their significance were calculated using the Pearson test, and comparisons of quantitative data performed by a paired sample t-test. A p-value of less than 0.05 was set as statistically significant.

## Results

In our study, 46 patients (mean age: 60.8 ± 10.2 years; gender: 28 male, 18 female) with moderate to very severe COPD undergoing PR and 32 healthy subjects (mean age: 58.5 ± 8.9 years; gender: 13 male, 19 female) were enrolled. Baseline characteristics are shown in Table 1. Age and sex distributions were similar between the two groups. According to Global Initiative for Chronic Obstructive Lung Disease (GOLD) classification; there were 22 class-II, 18 class-III, and 7 class-IV COPD patients. COPD patients had higher RV basal diameter, right atrial (RA) end-systolic area, sPAP, and MPI, as well as lower tricuspid annular plane systolic excursion (TAPSE) values compared to healthy control subjects in conventional echocardiographic measurements. In addition, there were significant differences in RV global LS and RV free wall LS between the two groups.

**Table 1:** Clinical, conventional echocardiographic, and ventricular strain data in patients undergoing pulmonary rehabilitation and in healthy control subjects

	Patients undergoing pulmonary rehabilitation (n: 46)	Healthy control subjects (n: 32)	p value
Age (years)	60.8 ± 10.2	58.5 ± 8.9	0.15
Gender (male,%)	28(61%)	13(41%)	
Body mass index (kg/m^2^)	28.2 ± 8.4	27.9 ± 7.2	0.67
Heart rate (beats/min)	78 ± 12	80 ± 10	0.77
GOLD classes 2/3/4, n	22/18/7		
RV end-diastolic basal diameter (mm)	38.1 ± 4.1	27.6 ± 3.5	< 0.001
RV end-diastolic longitudinal diameter (mm)	74.2 ± 9.4	60.4 ± 6.4	< 0.001
RV anterior wall thickness (mm)	4.35 ± 0.21	4.19 ± 0.31	0.82
RA end-systolic area (mm^2^)	17.2 ± 2.9	12.9 ± 1.8	< 0.001
sPAB (mmHg)	46.7 ± 15.4	24.8 ± 10.5	< 0.001
TAPSE (mm)	16.6 ± 2.6	20.4 ± 3.1	< 0.001
Tissue Doppler MPI	0.58 ± 0.08	0.35 ± 0.05	< 0.001
RV ejection fraction (%)	54.8 ± 4.9	56.3 ± 5.5	0.41
LV ejection fraction (%)	57.7 ± 5.5	59.4 ± 4.4	0.34
RV-TDI s'	12.9 ± 2.93	13.6 ± 3.06	0.38
RV free wall longitudinal strain (%)	18.1 ± 3.4	27.9 ± 3.6	< 0.001
RV global longitudinal strain (%)	20.4 ± 2.4	26.8 ± 3.2	< 0.001

Data are presented as mean ± standard deviation or percentile. Bold values indicate statistical significance p < 0.05. GOLD: global initiative for chronic obstructive lung disease; RV: right ventricle; RA: right atrium; sPAP: systolic pulmonary artery pressure; TAPSE: tricuspid annular plane systolic excursion; TDI s': tissue Doppler imaging systolic excursion; MPI: myocardial performance index; LV: left ventricle.

In post-rehabilitation echocardiography and 6MWT assessments, there were significant improvements in RV speckle-tracking measurements (Table 2) and increase in 6MWT. In 2DE measurements, there were differences among sPAP, TAPSE, and MPI. However, sPAP was only statistically significant.

**Table 2:** Standard echocardiographic and ventricular strain data in patients before and after pulmonary rehabilitation

	Before pulmonary rehabilitation (n:46)	3 months after pulmonary rehabilitation (n:46)	p value
RV end-diastolic basal diameter (mm)	38.1 ± 4.1	37.7 ± 4.0	0.23
RV end-diastolic longitudinal diameter (mm)	74.2 ± 9.4	73.5 ± 9.3	0.69
RV anterior wall thickness (mm)	4.35 ± 0.21	4.22 ± 0.26	0.87
RA end-systolic area (mm^2^)	17.2 ± 2.9	16.9 ± 2.4	0.18
sPAB (mmHg)	46.7 ± 15.4	43.2 ± 16.3	0.03
TAPSE (mm)	16.6 ± 2.6	17.2 ± 3.1	0.09
Tissue Doppler MPI	0.58 ± 0.08	0.55 ± 0.07	0.09
RV ejection fraction (%)	54.8 ± 4.9	55.2 ± 5.0	0.72
LV ejection fraction (%)	57.7 ± 5.5	57.4 ± 5.2	0.57
RV-TDI s'	12.9 ± 2.93	11.8 ± 3.06	0.47
RV free wall longitudinal strain (%)	18.1 ± 3.4	22.9 ± 3.7	< 0.001
RV global longitudinal strain (%)	20.4 ± 2.4	21.9 ± 2.9	< 0.001
Six-minute walk test (m)	326 ± 42.2	355 ± 57.1	< 0.001
mMRC score	1.8 ± 0.8	1.7 ± 0.7	0.14
BODE index	3.0 ± 2.1	2.8 ± 1.9	0.04

Data are presented as mean ± standard deviation. Bold values indicate statistical significance p < 0.05. RV: right ventricle; RA: right atrium; sPAP: systolic pulmonary artery pressure; TAPSE: tricuspid annular plane systolic excursion; MPI: myocardial performance index; LV: left ventricle; TDI s': tissue Doppler imaging systolic excursion; mMRC; modified medical research council, BODE; Body mass index, degree of Obstruction (FEV_1_), Dyspnea score (mMRC scale), Exercise capacity (six minute walk distance).

**Figure 2:**
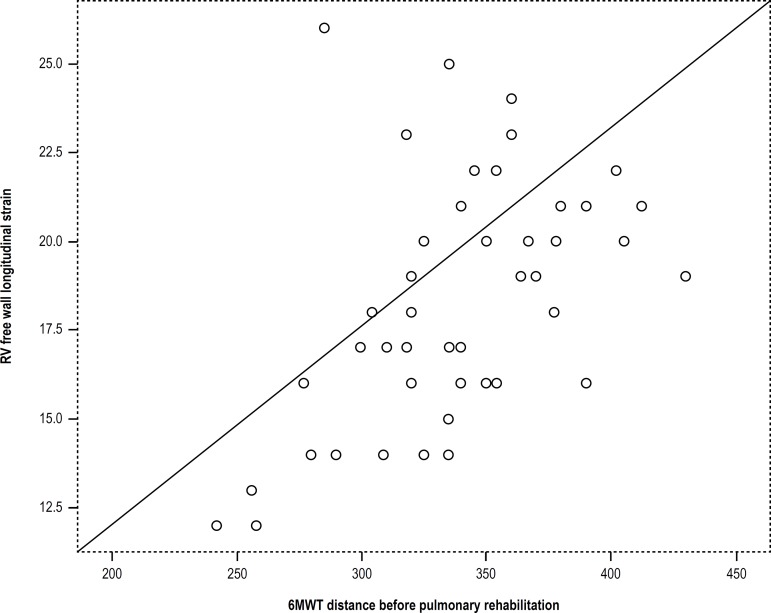
Correlation between right ventricular free wall longitudinal strain and six-minute walk test (6MWT) distance before pulmonary rehabilitation. (r = 0.58, p < 0.001). RV: right ventricular.

RV free wall LS was directly related to distance walked at baseline 6MWT (r = 0.58, p < 0.001) and to 6MWTDΔ (r = 0.41, p = 0.04) (Figura 2). There were improvements of both BODE index and MRC parameters, but only the BODE index was statistically different. There was a statistically significant correlation between RV free wall LS and BODE index (r: 0.52, p < 0.001).

## Discussion

In our study, we evaluated RV dysfunction in patients with moderate-to-very severe COPD in comparison with healthy subjects and also its improvement after PR program. In determination of both global and regional RV function improvement, STE was shown to be as effective as conventional 2DE. Moreover, RV global longitudinal strain was directly related to exercise tolerance determined by means of 6MWT and BODE index.

Although the current available prognostic models for COPD do not include RV function, it might serve as a surrogate endpoint for determining mortality and morbidity rates in a variety of cardiopulmonary diseases.^15,16^ On the other hand, RV assessment can be challenging. STE overcomes most of the limitations inherent in conventional 2DE, given that it is independent of cardiac translation, and it is angle- and load-independent, thus allowing accurate quantification of myocardial function.^17^ STE also demonstrates whether reduced RV performance is due to a global failure or to localized impaired contraction. Moreover, it identifies discrete and localized losses in contractility that are insufficient to affect global systolic function but have potential diagnostic and prognostic implications. The main result of the study by Focardi et al.^18^ was that free wall and global RV LS had a stronger correlation with the RV ejection fraction (RVEF) calculated by CMR (cardiac magnetic resonance) than conventional echocardiographic indices. Between the two, the highest diagnostic accuracy and the strongest correlation with the RVEF measured by CMR were observed for RV free wall longitudinal strain.^18^ In our study, RV free wall LS had higher improvement than RV global LS after PR program. Moreover, it had a statistically significant correlation with exercise tolerance indices of the patients, such as 6MWT distance and BODE index. One possible explanation for this is that the thin RV free wall contracts against low pulmonary resistance, thus leading to significantly higher strain improvement after the decline of pulmonary resistance by means of PR program. On the other hand, the septum consists of the same fibers as those forming the LV and must handle loading conditions in the RV, as well as higher LV afterload.16 Nevertheless, this hypothesis must be confirmed by further studies. In addition, we chose to analyze the septum as part of the RV. It cannot be considered simply a part of the LV because its shortening contributes to the ejection phase of the RV, and any impairment in its contractility reduces RV performance.^14,19^

Because of the paucity of data, no reference limits were established in the latest guidelines for RV global LS. Recent studies involving STE have focused on exploring RV function in patients with cardiopulmonary disease. Hardegree et al.^20^ showed that RV free wall LS and 6MWT distance were increased after the initiation of medical therapy in patients with pulmonary arterial hypertension (PAH). Motoji et al.^21^ showed that RV global LS < 19.4% indicates high risk of adverse cardiovascular events in patients with PAH. In addition, Guendouz et al.^22^ reported that an absolute RV global LS value below 21% in patients with congestive heart failure identifies patients with high risk of adverse cardiac events. However, to the best of our knowledge, there are no published studies using STE to determine RV dysfunction and its improvement after PR program in patients with COPD.

The effect of PR on RV function in patients with COPD has been explored in 2DE-based studies. Caminiti et al.^8^ showed that TAPSE ≤ 16 mm was an indicator of decreased 6MWT distance at baseline and 6MWT distance change in COPD patients undergoing PR. According to our study, STE was more sensitive in determining RV dysfunction than 2DE. Tanaka et al.,^23^ in another 2DE-based study, showed an increase of MPI, and that there was a strong correlation between MPI and the MRC breathlessness score in COPD patients.^23^ Our data are in agreement with these 2DE studies of RV function in COPD patients undergoing PR program.

### Study limitations

Several limitations of our study merit consideration. The main limitation was the small size of the study population. Moreover, RV strain was assessed only in the 4-chamber view of the six segments of the RV; however, the RV longitudinal function measured in the inlet chamber accounts for about 80% of RV function.^24^ If we had followed up the study population, we could have investigated the impact of PR program on RV function, as well as mortality and morbidity. Finally, we did not compare our results with those of CMR. However, previous studies of LV speckle tracking-derived strain have already validated CMR use. Furthermore, although magnetic resonance imaging is considered the gold standard for determining RV volume and function, it is currently limited by cost and availability and is deemed unsuitable after the implantation of a cardiac pacemaker.^25^

## Conclusion

Our study demonstrated that RV dysfunction improved after PR program in patients with COPD. STE might be as effective as the more established measurements of global RV function (i.e., TAPSE, RVEF, and MPI). RV global and regional strain assessment is a simple and effective tool in the routine clinical assessment of patients with COPD in order to explore the relationship between RV function and exercise tolerance.
